# Proteome-wide analyses of human hepatocytes during differentiation and dedifferentiation

**DOI:** 10.1002/hep.26414

**Published:** 2013-07-01

**Authors:** Cliff Rowe, Dave T Gerrard, Roz Jenkins, Andrew Berry, Kesta Durkin, Lars Sundstrom, Chris E Goldring, B Kevin Park, Neil R Kitteringham, Karen Piper Hanley, Neil A Hanley

**Affiliations:** 1 Centre for Endocrinology & Diabetes, Institute of Human Development, Faculty of Medical & Human Sciences, AV Hill Building, Manchester Academic Health Science Centre, University of ManchesterOxford Road, Manchester, UK; 2 Department of Pharmacology & Therapeutics, University of Liverpool, Sherrington BuildingAshton Street, Liverpool, UK; 3 MRC Centre for Drug Safety Science, University of Liverpool and University of ManchesterUK; 4 Bioinformatics, Faculty of Life Sciences, Michael Smith BuildingOxford Road, Manchester, UK; 5 Human Genetics Division, University of SouthamptonTremona Road, Southampton, UK; 6 SARTRE, School of Clinical Sciences, University of BristolBristol, UK; 7 Department of Endocrinology, Central Manchester University Hospitals NHS Foundation TrustOxford Road, Manchester, UK

## Abstract

Failure to predict hepatotoxic drugs in preclinical testing makes it imperative to develop better liver models with a stable phenotype in culture. Stem cell-derived models offer promise, with differentiated hepatocyte-like cells currently considered to be “fetal-like” in their maturity. However, this judgment is based on limited biomarkers or transcripts and lacks the required proteomic datasets that directly compare fetal and adult hepatocytes. Here, we quantitatively compare the proteomes of human fetal liver, adult hepatocytes, and the HepG2 cell line. In addition, we investigate the proteome changes in human fetal and adult hepatocytes when cultured in a new air-liquid interface format compared to conventional submerged extracellular matrix sandwich culture. From albumin and urea secretion, and luciferase-based cytochrome P450 activity, adult hepatocytes were viable in either culture model over 2 weeks. The function of fetal cells was better maintained in the air-liquid interface system. Strikingly, the proteome was qualitatively similar across all samples but hierarchical clustering showed that each sample type had a distinct quantitative profile. HepG2 cells more closely resembled fetal than adult hepatocytes. Furthermore, clustering showed that primary adult hepatocytes cultured at the air-liquid interface retained a proteome that more closely mimicked their fresh counterparts than conventional culture, which acquired myofibroblast features. Principal component analysis extended these findings and identified a simple set of proteins, including cytochrome P450 2A6, glutathione S transferase P, and alcohol dehydrogenases as specialized indicators of hepatocyte differentiation. *Conclusion*: Our quantitative datasets are the first that directly compare multiple human liver cells, define a model for enhanced maintenance of the hepatocyte proteome in culture, and provide a new protein “toolkit” for determining human hepatocyte maturity in cultured cells. (Hepatology 2013;58:799–809)

**H**epatotoxicity is a major contributor to drug failure during preclinical or clinical testing, or once licensed for patient use.^1^ In addition to enormous expense,^2^ it causes adverse events leading to patient morbidity and mortality.^3^ This demands more predictive *in vitro* models for use early during drug development alongside *in vivo* testing in rodents. Freshly isolated human primary adult hepatocytes are considered the “gold standard” for *in vitro* investigation of hepatocyte function and toxicity.^4^ However, they are difficult to source and nonproliferative in culture, necessitating the use of hepatocyte-like cell models. Hepatocellular cancer cells, especially the HepG2 line, are readily available and much used but they have failed to predict numerous hepatotoxic drugs.^5^ Hepatocyte-like cells derived from directly reprogrammed cells^6^ or pluripotent stem cells offer promise.^7^,^8^ For all these cells, *in vitro* application in toxicity-screening platforms is complicated by two issues: defining cell maturity, and refining culture methods so that they can be maintained long enough without loss of phenotype to be of practical use to the pharmaceutical industry. Although much is known about human adult hepatocytes, quantifying the first problem has been hampered by limited understanding of its fetal counterpart. The second reflects suboptimal culture and uncertainty over what is lost as phenotype deteriorates.^10^

Knowledge of the fetal hepatocyte phenotype is particularly important in the anticipation that terminal differentiation from stem cells will pass through a fetal-like phase^7^; or that when adult cells lose maturity in culture, it may represent dedifferentiation towards an embryonic state.^11^ However, direct comparison to fetal cells has commonly been lacking or assumed on the basis of a small number of transcript types or proteins, such as alfa-fetoprotein (AFP).^7^ In addition to the inference of cytochrome P450 (CYP) activity by reverse-transcription polymerase chain reaction, immunoblotting of microsomes, or *in vitro* metabolism of substrates,^12^–^15^ genome-wide transcript^16^ and proteome data^17^ are available on human fetal hepatocytes. However, these fetal data have chiefly been considered in isolation rather than alongside the equivalent information on adult hepatocytes or other human hepatocyte models, such as HepG2 cells.

Using conventional submerged culture in an extracellular matrix (ECM) sandwich,^18^ primary human adult hepatocytes dedifferentiate over the course of ∼1 week.^10^ As a first step towards prevention, understanding what protein changes underlie dedifferentiation would be helpful. Various 3D *in vitro* approaches have been proposed to enhance hepatocyte phenotype in culture. However, their value has commonly revolved around limited functions such as albumin secretion or individual CYP activity.^19^–^20^ Furthermore, many 3D systems are complex and unsuited to high-throughput platforms. Simple air-liquid interface (ALI) models may be better based on well-differentiated tissue-like cultures from other organ types.^21^–^22^

In this study, we deciphered the proteome of fresh human fetal hepatocytes compared quantitatively and directly against the equivalent data from human adult hepatocytes and HepG2 cells using isobaric tagging for relative and absolute quantification (iTRAQ) 8-plex labeling and mass spectrometry-based “shotgun” proteomics. Primary cell proteomes were reassessed after culture in a conventional submerged ECM-sandwich or at the ALI in 3D (ALI-3D). Principal components analysis (PCA) revealed combinations and levels of proteins that discriminated and defined each of the cell types including features acquired during dedifferentiation or maintained in ALI-3D culture. The combined data enhance definition of the hepatocyte phenotype and provide an evidence base for the use of ALI-3D culture.

## Materials and Methods

### Human Subjects and Tissue

Human fetal material was obtained from voluntary termination of pregnancy with informed consent under ethical approval as reported.^23^ Fresh human adult hepatocytes were obtained from Invitrogen (Warrington, UK). Collection, use, and storage of material followed the Codes of Practice of the Human Tissue Authority, UK. Fetal livers 1-8 were aged at 7, 7, 8, 8, 8, 9, 16, and 18.5 weeks postconception (wpc), respectively. Further details are in the Supporting Text.

### Immunohistochemistry and Immunoblotting

Immunohistochemistry or immunoblotting for sex-determining region Y box 9 (SOX9), multidrug resistance protein 2 (MRP2), cytochrome P450 (CYP) 1A1/1A2, CYP2D6, CYP3A4/3A7, CYP2A6, glutathione S transferase π (GSTp), and heat shock protein 47 (HSP47) were conducted as reported^23^ or as described in the Supporting Text.

### Protein Isolation and Proteomic Analysis

Tissue or cell homogenates, prepared in triethylammonium bicarbonate buffer/0.1% sodium dodecyl sulfate (TEAB/SDS) by sonication, were clarified by centrifugation (10,000*g*, 2 minutes) and supernatant protein concentration determined by Bradford assay. 8-plex iTRAQ reagent labeling (Applied Biosystems) was carried out according to the manufacturer's instructions using 100 μg protein for each sample (see Supporting Text). Sample complexity was reduced by strong cation exchange and fractions of 2 mL were collected. Liquid chromatography (LC) followed by tandem mass spectrometry (MS/MS) of peptide-rich fractions was performed on a QSTAR Pulsar I hybrid mass spectrometer (AB Sciex). To avoid bias from interrun variation, iTRAQ labeling was varied for the fresh adult hepatocyte samples, which were spread across the four proteomics experimental runs. In addition, each run included a common reference preparation consisting of pooled samples from the different experiments. Quantitation of proteins was relative to this common pooled sample. Proteins identified by two or more peptides with at least 90% confidence, or by a single peptide with at least 99% confidence, were included in subsequent analyses.

### Cell Culture

HepG2 cells were sourced and cultured as reported.^23^ Human fetal hepatocytes and human adult hepatocytes were cultured in Williams' E medium supplemented with 2 mM L-glutamine, insulin-transferrin-selenium (ITS), and 100 nM dexamethasone at 5% CO_2_ and 37°C. Cells were seeded onto Matrigel-coated 6-well plates (1.5 million cells per well) overlaid 3 hours later with a second layer of Matrigel (ECM-sandwich) or onto a single ALI-3D membrane in each well of a 6-well plate. ALI-3D membranes are hydrophilic polytetrafluoroethylene (PTFE) membrane discs (6 mm diameter, 0.4 μm thick; BioCell Interface SA). These were placed onto a 0.4 μm Millicell-standing cell culture plate insert (Millipore, UK) and 1.5 mL media added beneath the insert. Media were exchanged every 2 days for the duration of the primary cultures until 15 days and retained for analysis.

### Albumin Secretion, CYP3A Activity, and Urea Output Assays

The secretion of albumin and urea into media samples was determined 1 day after culture initiation and every 2 days thereafter using a human albumin enzyme-linked immunosorbent assay (ELISA) kit (Bethyl Laboratories) and QuantiChrom urea assay kit (Bethyl Laboratories). CYP3A activity was assessed in duplicate by incubation with P450-Glo CYP3A4 Assay reagent (Luciferin-PFBE; Promega).

### Alcohol Dehydrogenase Activity

Samples were homogenized in 50 mM sodium pyrophosphate buffer (pH 8.8) by pipetting and sonication, and clarified by centrifugation at 14,000 rpm for 1 minute. The reduction of nicotinamide adenine dinucleotide (NAD) to NADH during the catalysis of ethanol to acetaldehyde was measured by increase in absorbance at 340 nM by spectrophotometry over 5 minutes according to the protocol from Sigma-Aldrich.

### Bioinformatics and Statistics

Protein quantities, relative to the common reference pool, were used as input when comparing proteomes. The relationships between samples were inspected as a heatmap using Euclidean distance for proteins common to all samples. PCA was conducted using this set of proteins. Proteins were annotated with gene ontology (GO) terms and tested for enrichment in two ways: first, using GO terms detected in all samples against the full list of human proteins with GO annotations (“detection test”: see Supporting Text for details); and second, using protein-specific scores from selected PCs to test for association of extreme scores with GO terms (“PC score tests”: see Supporting Text for details). All analyses were conducted in R (v. 2.11.1) with the assistance of “lattice,” “gplots,” “qvalue,” and “org.Hs.eg.db” R packages. R scripts are available at https://github.com/davetgerrard/LiverProteins.

## Results

### Human Fetal Liver Demonstrates Aspects of Maturity During Early Development

**Figure 1:**
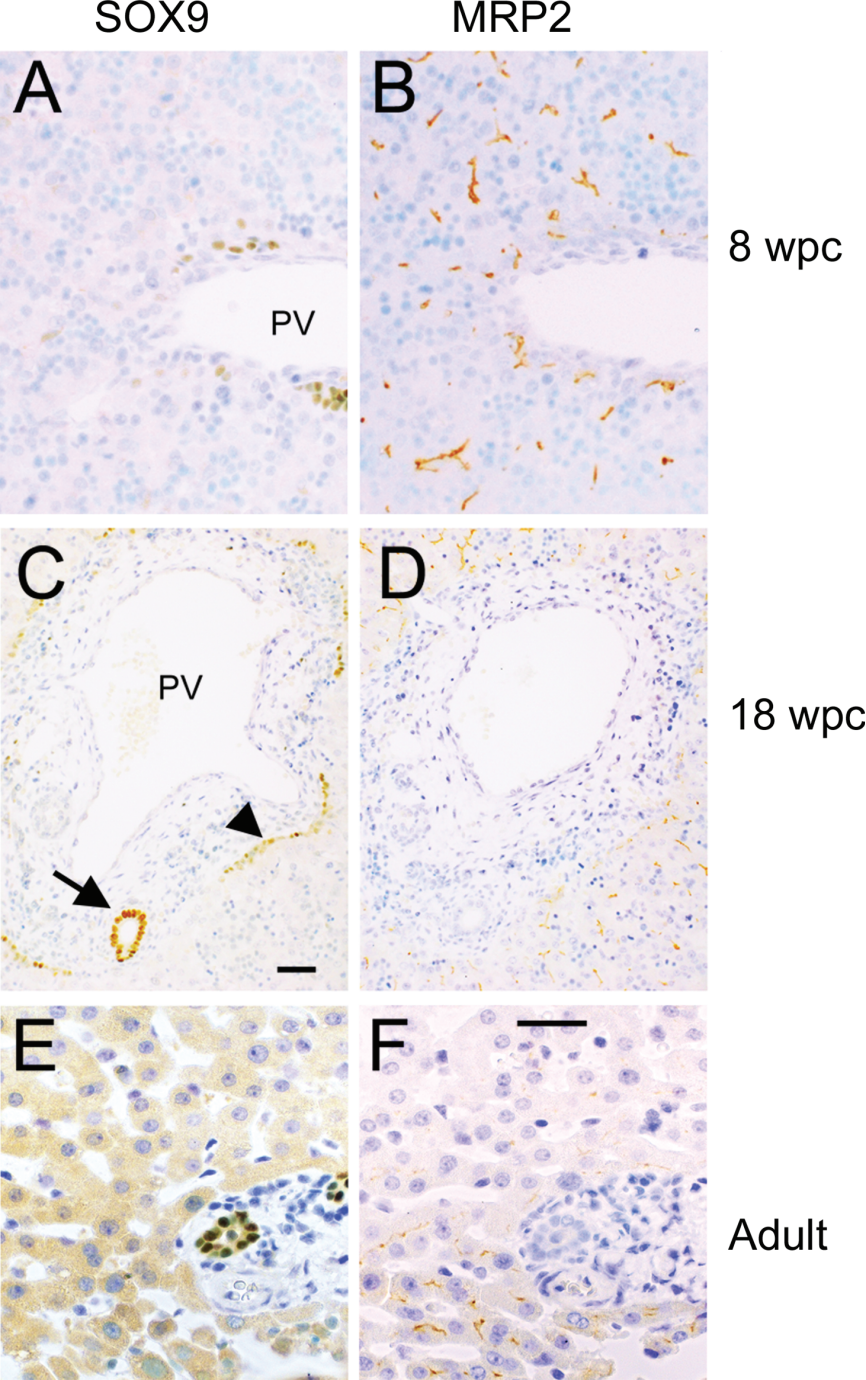
. Detection of SOX9 and MRP2 in human fetal and adult liver. Brightfield immunohistochemistry (brown) marks the ductal plate and bile ducts (SOX9) and canalicular organization (MRP2) counterstained with Toluidine blue in fetal liver at 8 wpc (A,B), 18 wpc (C,D), and in adult liver (E,F). The arrow indicates an intrahepatic bile duct and the arrowhead indicates the ductal plate (C). PV, portal vein. Scale bars = 200 μm (A-D;E-F).

**Figure 2:**
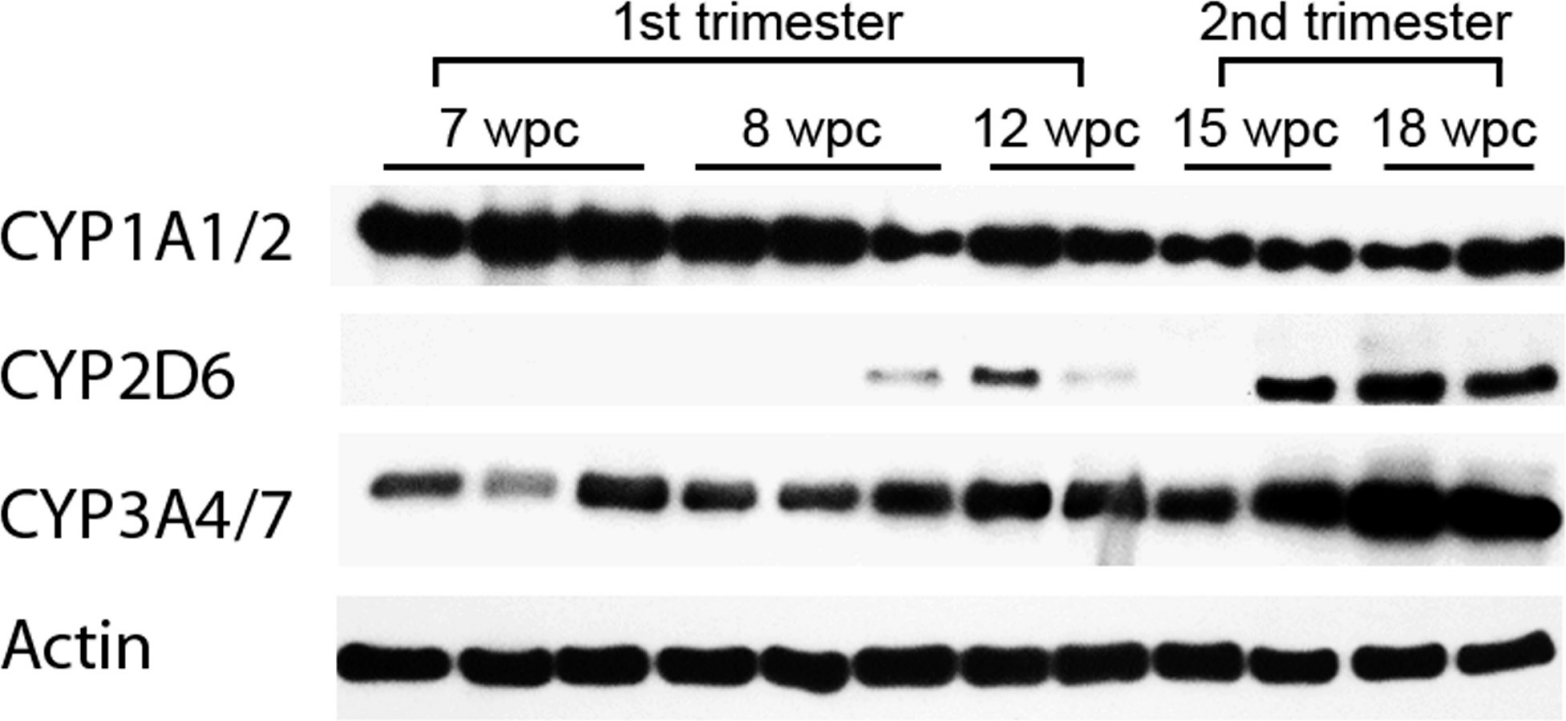
. Immunoblotting for CYPs during the first two trimesters of liver development. Immunoblotting for CYP1A1/2, CYP2D6, and CYP3A4/7 is shown at various stages during the first and second trimester.

To guide subsequent proteomic analyses, immunohistochemistry was undertaken to indicate when hepatocytes organize and function in the fetal liver. Encircling the portal vein, the ductal plate, which gives rise to periportal hepatocytes and intrahepatic bile ducts,^24^–^25^ could be discerned at 8 wpc by nuclear SOX9 (Fig. 1A).^26^ The ductal plate was more organized in the second trimester (Fig. 1C). At 8 wpc, organized periluminal MRP2 was detected in fetal hepatocytes indicating the formation of bile canaliculi and polarization of fetal hepatocytes (Fig. 1B), at which point CYP1A1/A2 and CYP3A4/A7, but not CYP2D6, were apparent by immunoblotting (Fig. 2). Although hepatic CYP2D6 expression is known to vary among individuals,^27^ it could be detected in individual human fetal liver samples by the end of the first trimester, with more robust detection in older specimens (Fig. 2).

### Human Fetal Hepatocytes Are Maintained Better in ALI-3D Than in ECM-Sandwich Culture

**Figure 3:**
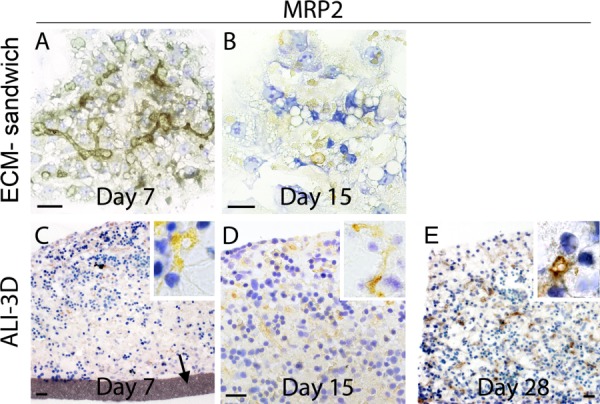
. Culture of human fetal hepatocytes in ECM-sandwich and ALI-3D format. As an indication of epithelial cell polarity and bile canaliculus formation in ECM-sandwich and ALI-3D cultures, immunolocalization of MRP (brown) is shown after 7 days (A,C) and 15 days (B,D) of culture counterstained with Toluidine blue. Organized MRP2 staining was lost at 15 days in ECM-sandwich format. (E) Periluminal canalicular MRP2 staining was still present in human fetal liver in ALI-3D format after 28 days of culture. Insets (C-E) show higher-magnification examples of MRP2-stained canaliculi. Arrow shows the ALI-3D membrane (C). Scale bars = 100 μm.

**Figure 4:**
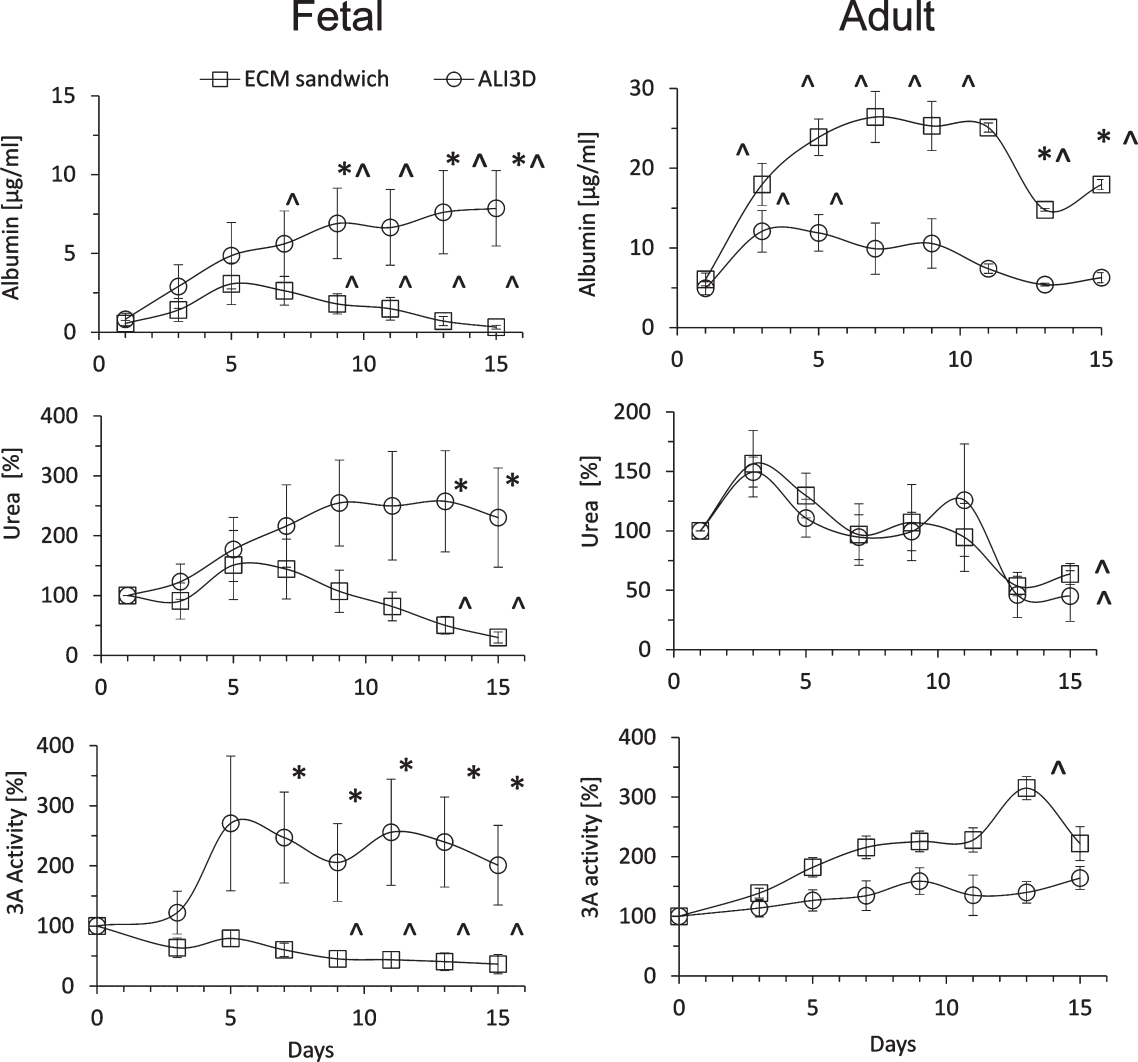
. Albumin and urea secretion and CYP3A activity of cultured human hepatocytes. Measurements were made every 2 days during ECM-sandwich and ALI-3D culture for albumin and urea secreted into the media and CYP3A activity. *P < 0.05 compared with the equivalent data point of the other culture method; *P* < 0.05 compared with first data point of the same culture method.

To provide *in vitro* models for downstream proteomic assessment, we compared standard ECM-sandwich versus ALI-3D culture on a semipermeable membrane. Human fetal hepatocytes in ECM-sandwich culture retained MRP2 as a marker of polarity and bile canaliculi for 1 week (Fig. 3A). However, by 2 weeks organized MRP2 staining was lost and a lot of cell death had occurred (Fig. 3B). In comparison, human fetal liver in ALI-3D format retained periluminal MRP2 expression for 1 month (Fig. 3C-E). Monolayer and ALI-3D fetal cells initially secreted similar amounts of albumin and urea, and possessed similar CYP3A activity (Fig. 4). All three parameters were maintained or rose in ALI-3D culture over 2 weeks. In ECM-sandwich culture, values for albumin, urea, and CYP3A activity were maintained for 5 days but thereafter they declined. For adult cells, albumin secretion rose statistically during ECM-sandwich culture and was significantly higher than corresponding values from ALI-3D culture after 2 weeks (Fig. 4). Levels of urea secretion in both types of adult cell culture were maintained relatively similarly, although they diminished slightly at later timepoints. CYP3A activity was maintained in both adult formats, with some statistical evidence of increasing values during ECM-sandwich culture. In comparison to the starting fetal samples, initial albumin secretion was ∼5-fold higher and urea secretion approximately 10-fold higher (100 mg/mL versus 10 mg/mL) in adult cells.

### Proteomes of Fresh Human Fetal Hepatocytes, Adult Hepatocytes, and HepG2 Display Distinct Hierarchical Clustering That Is Preserved After Culture

**Figure 5:**
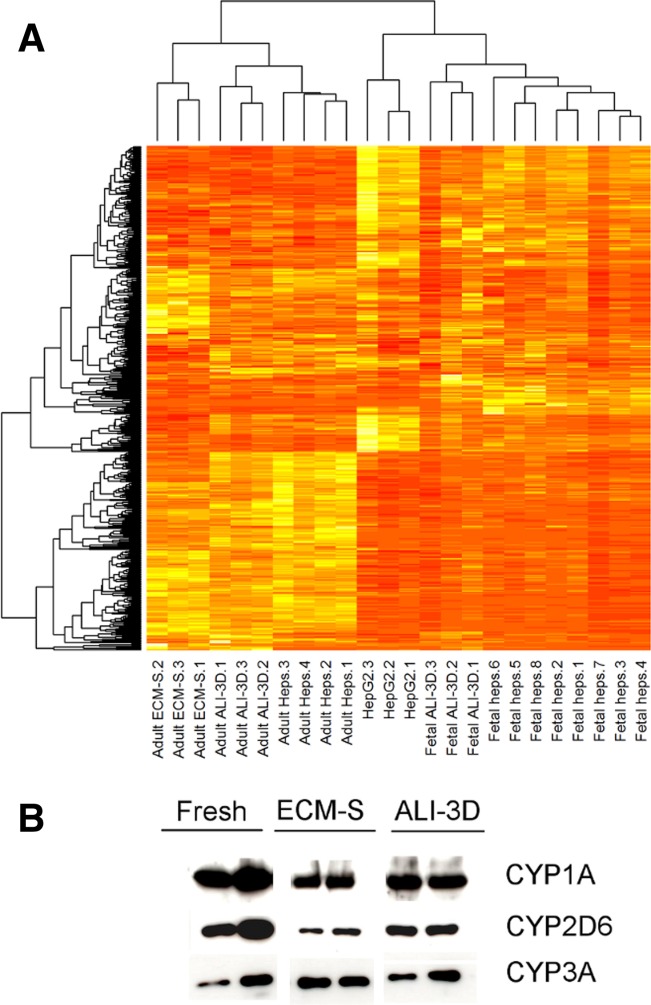
. Proteomic data from fresh and cultured human adult and fetal hepatocytes and HepG2 cells. (A) Heatmap generated from 432 proteins detected in all samples by hierarchical clustering using paired Euclidean distance. The mean quantitative value across all proteins was equal for all samples indicating equivalent total protein labeling. (B) Immunodetection of selected CYPs in adult hepatocytes in both culture systems and in fresh cells. ECM-S, ECM-sandwich.

To compare human fetal hepatocytes with fresh human adult hepatocytes and HepG2 cells, we conducted proteomic analyses of at least three and up to eight samples from each group. All primary samples came from different fetuses (7-18.5 wpc) or donors. In addition, we analyzed cultured primary cells from the same “parent” samples with the hypothesis that the proteome post-ALI-3D culture would more closely mimic the starting cell type than following ECM-sandwich culture. Fetal ECM-sandwich samples had to be excluded because they were so deteriorated after 15 days that sufficient protein could not be retrieved. The data may be downloaded from the ProteomeCommons.org Tranche repository using the following hash: 9/QzhS9LTd3uhbtbDlyhL9MG+EXiVHKRS3raaJ74nbmjjr7qNYS54V7BirAVnLeVFGrXsgGlY2IjYiGu5kdZwQcDpz8AAAAAAAAxWA==. A total of 1,507 different proteins were identified across four iTRAQ labeling and LC-MS/MS experiments after applying a cutoff for the 1% false discovery rate in any one experiment. The raw protein quantification data for each experiment and a summary sheet for the combined experiments are supplied (Supporting Dataset 1). Initial analysis sought to determine whether any protein(s) could uniquely distinguish any of the sample groups, i.e., proteins that were specific to fetal or adult hepatocytes, HepG2 cells, or either culture condition. However, strikingly, no protein was uniquely indicative of cell-type by being present only in one particular group and absent from all other samples. In fact, proteins were consistently detected in some samples from every group, indicating a major phenotypic overlap between human fetal and adult hepatocytes and HepG2 cells. To explore this further and to avoid any correlation between protein detection and experimental run, we filtered the dataset for proteins that were quantified in every sample. There were 432 such proteins. Statistical analysis of their associated GO terms revealed enrichment of core hepatocyte roles such as xenobiotic metabolism, oxidoreductase activity, and gluconeogenesis (Supporting Dataset 2). Using this dataset, the relationship between different samples was inspected as a heatmap (Fig. 5A). The order of samples was determined by hierarchical clustering using paired Euclidean distance. Cosegregation occurred entirely according to sample type with a clear division between adult-derived samples and the fetal samples that coclustered with HepG2. Fetal samples did not cluster according to developmental age. Thus, quantitatively, human fetal liver, adult hepatocytes, and HepG2 cells possessed discrete proteomes. Furthermore, samples clustered according to culture method, and directly adjacent to their associated fresh cell type. Thus, apart from the degraded fetal ECM-sandwich specimens, there was recognizable retention of proteome following culture for 2 weeks. However, the ALI-3D method was consistently closer to fresh tissue than ECM-sandwich, illustrating a more comprehensive retention of the fresh hepatocyte phenotype than was discernible from the albumin, urea, and CYP3A datasets (Fig. 4). These findings correlated to better retention of fresh levels of CYP1A and CYP2D6 immunoreactivity in ALI-3D format after 15 days than in ECM sandwich (Fig. 5B). Conversely, CYP3A immunoreactivity (detecting both CYP3A7 and CYP3A4) appeared more readily detected following ECM sandwich culture, consistent with the iTRAQ quantification for CYP3A4 (Fig. 5B; Supporting Dataset 1).

### PCA Defines Characteristics for Different Hepatocytes and Demonstrates Proteomic Markers for Dedifferentiation in ECM-Sandwich Versus Maintenance of Function in ALI-3D Culture

**Figure 6:**
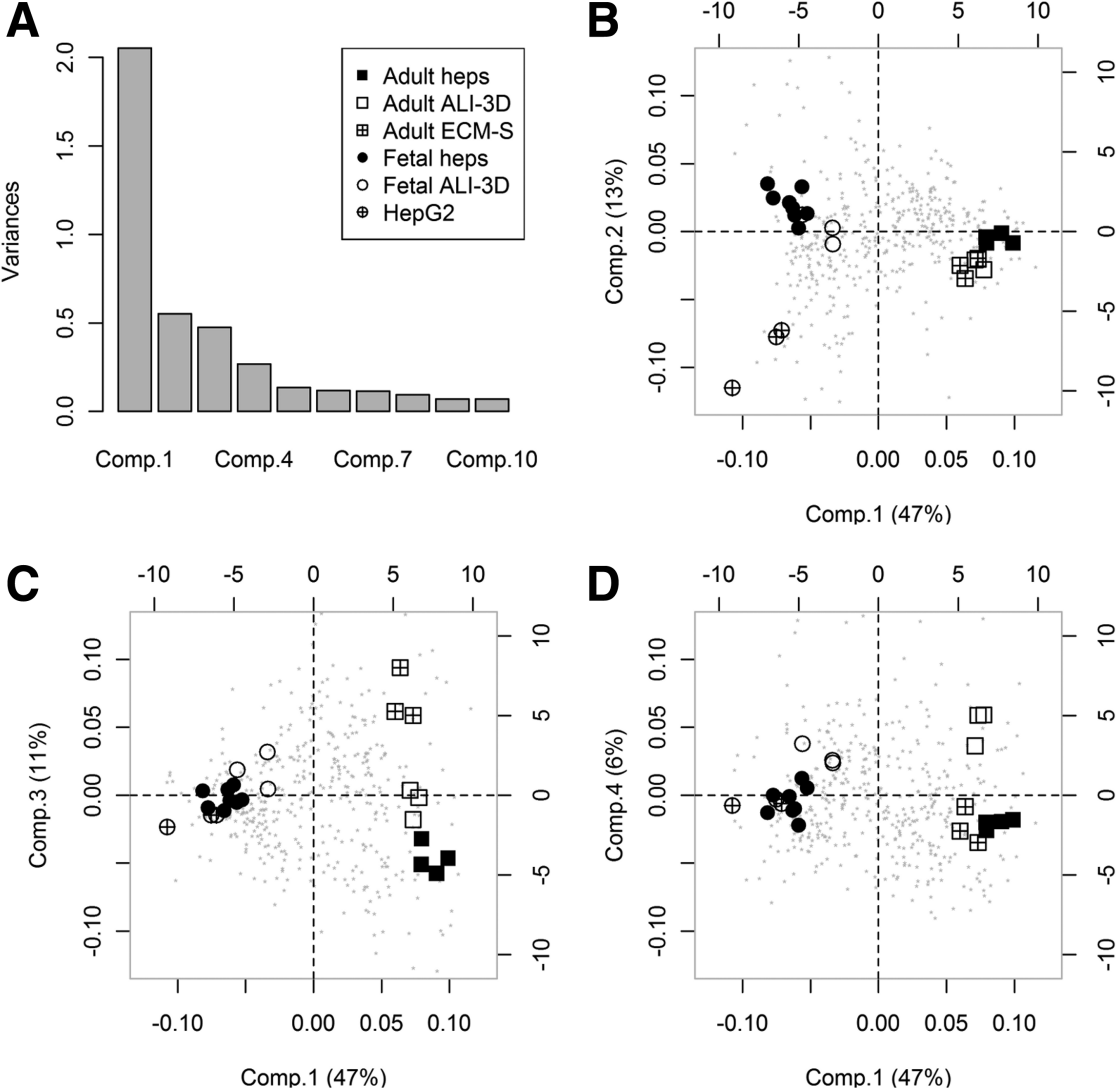
. PCA of proteomic data from fresh and cultured human adult and fetal hepatocytes and HepG2 cells. (A) Variance contributed to the analysis by each of the first 10 PCs. The remaining 14 PCs each captured <1% of the variance and are not shown. (B-D) PC2 (B), PC3 (C), and PC4 (D) are shown plotted on the y axis against PC1 on the *x* axis. The loadings of each sample on each pair of PCs are enumerated in the bottom and left axes. The scores of individual proteins, projected onto each PC, are marked as gray points enumerated in the top and right axes.

**Figure 7:**
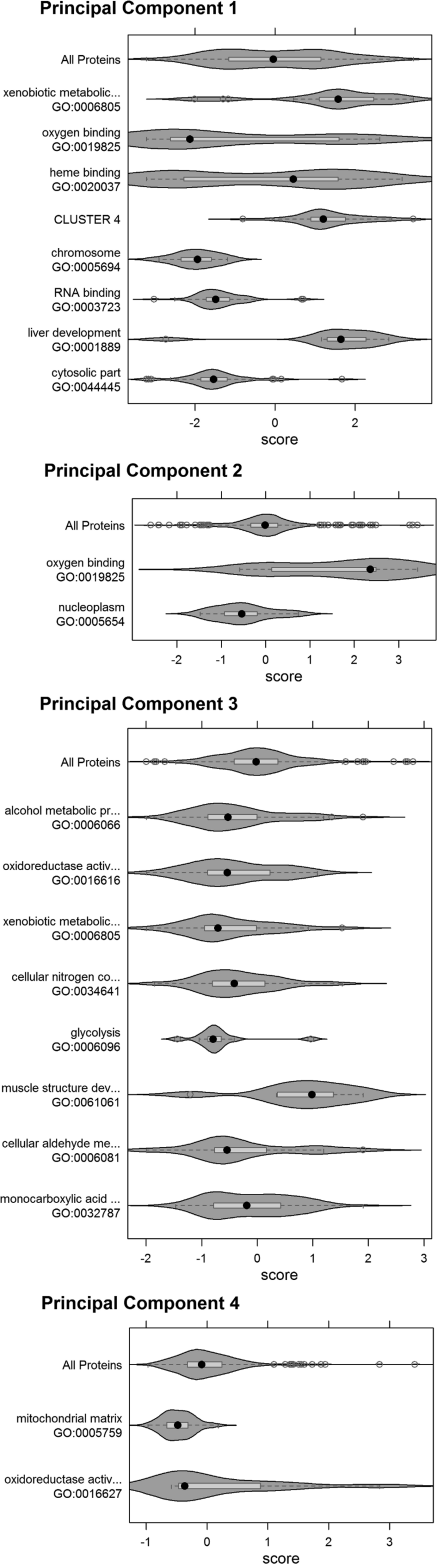
Violin plots of enriched GO terms for principal components 1 to 4. All GO terms with unadjusted *P* < 0.001 are included following the elimination algorithm and the Wilcoxon test on absolute PC scores (elimAbsWilcox, see Materials and Methods). The full distribution of scores for each PC is given under “All Proteins.” Black dots represent the median value of each set of proteins. The violin plots indicate the PCA scores associated with specific GO terms. “CLUSTER 4” represents a set of proteins that overlap strongly with multiple highly significant GO terms pertaining to ribosomal function. The equivalent figures using the standard Wilcox test and the Kolmogorov-Smirnov tests are shown in Supporting Figs. 2 and 3, respectively. The PCA scores for each protein are shown in Supporting Dataset 3.

Having identified that cells in ALI-3D format clustered hierarchically closer to fresh cells than cells in ECM-sandwich culture, we wanted to detect which of the 432 proteins contributed to these characteristics. We utilized PCA (the first four principal components are provided in Supporting Dataset 3), and performed GO term analyses based on PCA scores. The GO term analysis was statistically examined by absolute Wilcoxon ranks sum test (Fig. 6; and Supporting Dataset 4) with corroboration by two other statistical tests: Wilcoxon (using untransformed scores) and Kolmogorov-Smirnov tests (further details in Supporting Text). The first four principal components capture 77% of the variance in the dataset; after the first 10, PCs were individually responsible for <1% and not analyzed further (Fig. 6A). Figure 6B-D shows the proportion of variance contributed by each sample to the first four PCs. The greatest component of variation in the data, PC1 (47% of the variance), clearly differentiated all adult hepatocyte from all fetal and HepG2 samples; the latter two groups were located with similar negative PC1 scores. When grouped by GO terms the proteins giving rise to the PC1 score for adult hepatocytes were identified by “xenobiotic metabolic process” and “liver development,” displayed as violin plots in Fig. 7 (violin plots using the alternative statistical tests are in Supporting Figs. 1 and 2). A positive PC2 score and negative PC1 score discriminated fresh human fetal hepatocytes. Individually listing the 75 proteins with the most negative PC1 scores and most positive PC2 scores allowed 15 common proteins to be identified, including AFP and CYP3A7, that discriminated the fresh fetal hepatocyte phenotype from that of either adult hepatocytes or HepG2 cells (Supporting Table 1). A negative score for PC1 and PC2 (which contributed 13% of the variance) very clearly distinguished HepG2 cells, represented on the violin plots by the GO term “nucleoplasm.” From the 75 proteins with the most negative PC1 and PC2 scores, 25 were in common to discriminate HepG2 cells (Supporting Table 1).

For PC3 (11% of the variance in the dataset; Fig. 6C), adult hepatocyte proteins with positive scores were enriched in samples cultured in ECM sandwich, whereas the fresh adult samples all had negative PC3 scores. This correlated on GO analysis to the coordinated loss of proteins in ECM-sandwich culture that comprise key liver metabolic functions such as “glycolysis,” “xenobiotic metabolic process,” and “alcohol metabolic process”; accompanied by an increase in structural proteins that map to the “muscle structure development” GO term (Fig. 7). In fact, up-regulated proteins after ECM-sandwich culture, such as myosin light polypeptide 6, had myofibroblast cytoskeletal or cell migration roles.^28^ Although surprising but consistent with Fig. 5B, CYP3A4 was also identified (Supporting Table 2). In contrast, by analyzing the 75 proteins with the most negative PC3 and most positive PC1 scores, a common subset of 28 proteins could be identified that best characterized fresh human adult hepatocytes including three CYPs and four alcohol dehydrogenases (ADHs; Supporting Table 1).

### Immunoblotting and Functional Analysis Verify PCA Identified Hepatocyte Maturity Markers

**Figure 8:**
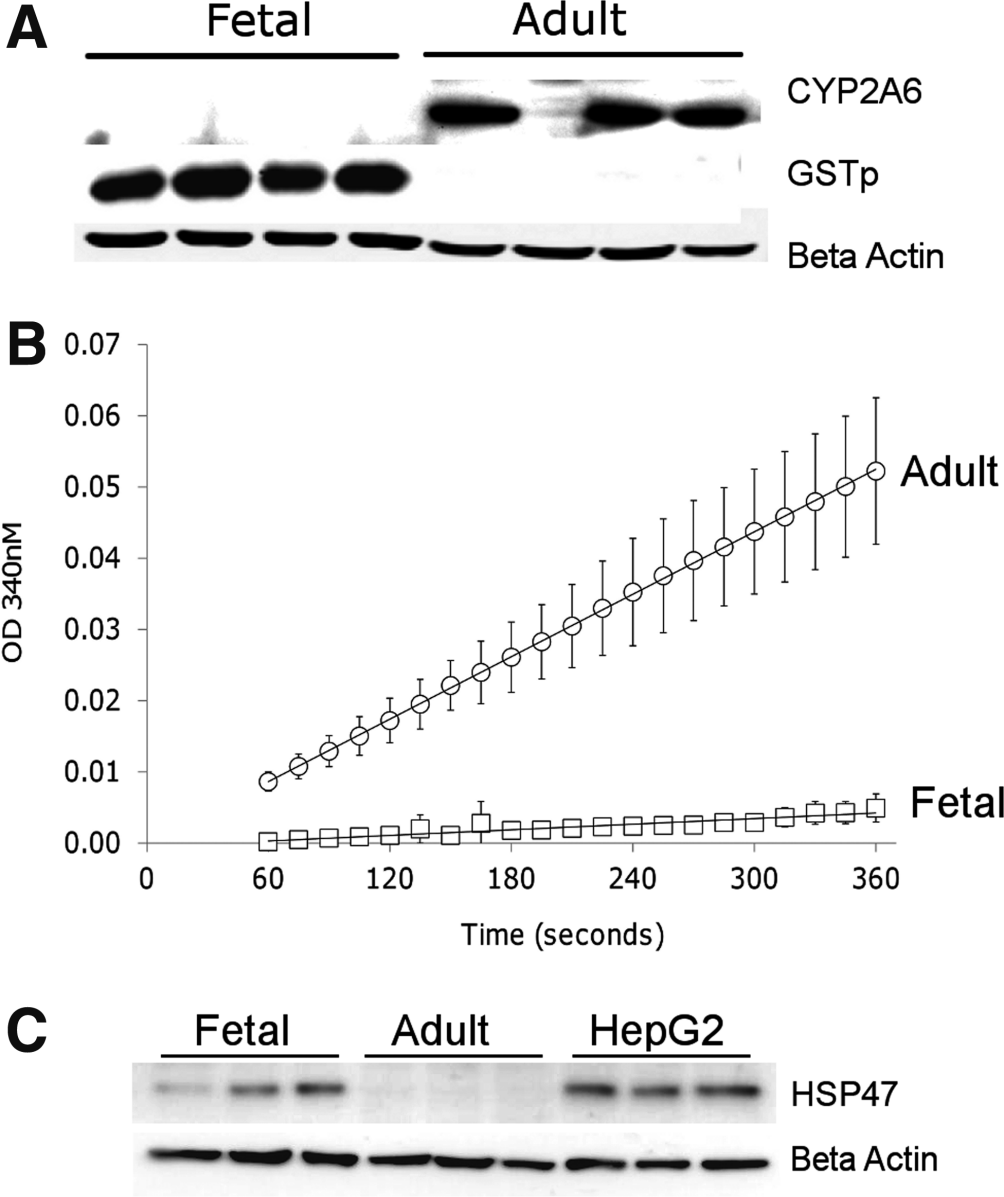
. Immunoblot and functional analysis verify PCA-identified hepatocyte maturity markers. (A) Immunoblot analyses of CYP2A6, GSTp, and β-actin in four samples each of adult hepatocytes and fetal liver. (B) Alcohol dehydrogenase activity measured by formation of NADH in three adult hepatocyte preparations (circles) and three fetal liver samples (squares). Data are presented as mean Abs340; error bars indicate the ± SD. (C) Immunoblot analyses of heat shock protein 47 (HSP47) and β-actin in three samples each of fetal and adult hepatocytes and HepG2 cells.

To verify the PCA that implied discriminatory proteins for adult, fetal, and HepG2 hepatocytes, we performed selected immunoblotting and functional analysis. CYP2A6 was strongly detected in three out of four adult samples (Fig. 8A), consistent with the iTRAQ quantification where one sample had fetal-like levels of CYP2A6. All adult samples weakly expressed GSTp. Conversely, fetal samples showed robust GSTp and weak or absent CYP2A6 (Fig. 8A). The clear linear increase in absorbance for adult hepatocyte soluble protein samples indicated ADH activity, whereas fetal-derived protein barely catalyzed the reaction (Fig. 8B). Finally, immunoblot for heat shock protein 47 illustrated the predicted detection in fetal and HepG2 cells, but low levels in adult hepatocytes (Fig. 8C).

## Discussion

How best to maintain a mature cell phenotype is a challenge that confronts the culture of all primary cell-types. For cell-types generated from stem or progenitor cells, there is also the expectation that a terminally differentiated cell will have progressed through a fetal-like stage. To assess liver cell phenotype, previous unbiased datasets were mostly of transcripts until the Human Liver Proteome Project (HLPP) reported on 6,788 proteins from adult liver and 2,485 proteins from fetal liver.^17^,^29^ Our data complement the HLPP in two ways. First, rather than detail proteomes individually, we directly compared fetal and adult cells alongside a pharmaceutical toxicology “standard,” the HepG2 cell, to provide a differential proteome signature. Second, we used these data to identify changes underlying dedifferentiation of human hepatocytes in conventional culture and how this might be overcome by revised methodology. Our approach extended the novelty of using a common reference pool to expand the eight channels available in a single iTRAQ experiment by allowing integration of multiple experiments.^31^ The clustering of samples within groups by heatmap validates the technique, which is limited only by the availability of the common pool. Previous proteome studies have compared primary mouse hepatocytes against a mouse liver cell line,^32^ the dynamic phenotype of cultured rat hepatocytes,^33^ or changes in the HepG2 proteome in response to hepatotoxins.^34^ A comparison of primary human hepatocytes, HepG2 cells, and Hep3B cells identified 16 proteins as exclusive to hepatocytes.^35^ Interestingly, we detected 10 of these proteins across all hepatocyte types in our study. However, in each case the proteins were more abundant in primary adult cells than HepG2 cells and two, Arginase-1 and Fructose-bisphosphate aldolase B, predict the fresh adult phenotype by PC score (Supplementary Table 1).

Fetal hepatocytes possessed a range of CYP enzymes that catalyze phase I drug metabolism and, among the proteins detected in all eight fresh human fetal liver samples, more than 15 transferase enzymes with roles in phase II metabolism (Supporting Dataset 1). We found no proteins that were expressed exclusively in any sample type, demonstrating that AFP is not by itself indicative of a fetal phenotype. Similarly, differences in albumin secretion by adult and fetal cells were modest and by iTRAQ quantification, the protein was detected as readily in each cell-type. Furthermore, CYP3A4, commonly used to imply mature hepatocyte function of stem cell derivatives,^36^ was only 2.6-fold increased in adult hepatocytes compared to fetal cells; antibodies for immunoblotting fail to discriminate it from CYP3A7 (2.5-fold increased in fetal cells); commercial CYP3A4 luciferase assays demonstrate significant crossreactivity; and by PC scores CYP3A4 actually characterized suboptimal ECM-sandwich culture. However, PCA did allow new listings of proteins, which when abundant in combination discriminated fetal and adult hepatocytes and HepG2 cells from each other, thus offering revised determinants of hepatocyte maturity. For example, CYP2A6 was 5.75-fold more abundant in adult hepatocytes compared with fetal liver. PCA of our proteomics and validation tests showed that various ADHs underlying extreme PC1 and PC3 scores clearly discriminated fresh adult cells, consistent with the known increase in expression of *ADH* isoforms during liver development.^14^–^37^ Conversely, the PCA data and immunoblotting demonstrated GSTp (3.03-fold increased in fetal cells) coupled with low ADH activity was a robust indicator of the fetal liver phenotype. Interestingly, C/EBPα is known to promote expression of *ADH* isoforms^38^ and *CYP2A6*^39^ while suppressing *GSTp* expression,^40^ whereas various hepatocyte nuclear factor (HNF) family members also upregulate *ADH4* and *CYP2A6*.^39^–^41^ Thus, collectively these findings imply that the activity of these transcription factors is important in the transition from a fetal to an adult phenotype. The protein with the lowest PC1 score and positive PC2 score that segregated human fetal liver samples on PCA was myristoylated alanine-rich C-kinase substrate (MARCS). MARCS has been implicated in the regenerative nodules of cirrhosis^42^ but has not previously been described in human fetal liver.

Dedifferentiation of human hepatocytes in culture is problematic. In this study, albumin and urea secretion, and CYP3A activity, all commonly used markers of hepatocyte function, demonstrated better retention of cell phenotype for fetal hepatocytes in ALI-3D culture compared to ECM sandwich. However, for adult cells this information was only gained by the full proteomic analysis, which demonstrated that loss of the adult phenotype was not reversion to a fetal-like progenitor proteome. In contrast, it involved acquisition of a myofibroblast phenotype, consistent with epithelial-to-mesenchymal transition (EMT).^28^ Others have recently implicated repression of EMT as key to retaining the hepatocyte phenotype via HNF4α-mediated inhibition of Snail.^43^–^44^ Myofibroblastoid characteristics, such as myosin components, were not prominent in our ALI-3D model, which allowed retention of fetal bile canaliculi for 1 month. We postulate that increased oxygenation and enhanced multicellular architecture in the ALI-3D culture produce a more physiological microenvironment for hepatocytes than in the ECM sandwich culture.

In summary, these data provide the first comparative quantitative proteomic analysis of human adult and fetal hepatocytes and HepG2 cells, thereby providing a framework for informed judgment of future stem cell-derived hepatocyte-like cells beyond transcriptomic datasets. They also demonstrate proteomic changes that typify dedifferentiation of hepatocytes in ECM-sandwich culture, contrasted to a new culture method which better maintains hepatocyte phenotype and function.

## Abbreviations

ALI-3D: air-liquid interface-3-dimensional
CK19: cytokeratin 19
CYP: cytochrome P450
ECM: extracellular matrix
GO: gene ontology
GSTp: glutathione S transferase π
HNF: hepatocyte nuclear factor
iTRAQ: isobaric tagging for relative and absolute quantification
MRP2: multidrug resistance protein 2
PCA: principal components analysis
SOX9: Sex-determining region Y box 9
wpc: weeks post conception.

## References

[b1] Lee WM (2003). Acute liver failure in the United States. Semin Liver Dis.

[b2] Watkins PB (2011). Drug safety sciences and the bottleneck in drug development. Clin Pharmacol Ther.

[b3] Kola I, Landis J (2004). Can the pharmaceutical industry reduce attrition rates?. Nat Rev Drug Discov.

[b4] Soars MG, McGinnity DF, Grime K, Riley RJ (2007). The pivotal role of hepatocytes in drug discovery. Chem Biol Interact.

[b5] Castell JV, Jover R, Martinez-Jimenez CP, Gomez-Lechon MJ (2006). Hepatocyte cell lines: their use, scope and limitations in drug metabolism studies. Expert Opin Drug Metab Toxicol.

[b6] Huang P, He Z, Ji S, Sun H, Xiang D, Liu C (2011). Induction of functional hepatocyte-like cells from mouse fibroblasts by defined factors. Nature.

[b7] Baxter MA, Rowe C, Alder J, Harrison S, Hanley KP, Park BK (2010). Generating hepatic cell lineages from pluripotent stem cells for drug toxicity screening. Stem Cell Res.

[b8] Kia R, Sison RL, Heslop J, Kitteringham NR, Hanley N, Mills JS (2013). Stem cell-derived hepatocytes as a predictive model for drug-induced liver injury: are we there yet?. Br J Clin Pharmacol.

[b9] Sison-Young RL, Kia R, Heslop J, Kelly L, Rowe C, Cross MJ (2012). Human pluripotent stem cells for modeling toxicity. Adv Pharmacol.

[b10] Elaut G, Henkens T, Papeleu P, Snykers S, Vinken M, Vanhaecke T (2006). Molecular mechanisms underlying the dedifferentiation process of isolated hepatocytes and their cultures. Curr Drug Metab.

[b11] Lee SJ, Friedman SL, Whalen R, Boyer TD (1994). Cellular sources of glutathione S-transferase P in primary cultured rat hepatocytes: localization by in situ hybridization. Biochem J.

[b12] Johnsrud EK, Koukouritaki SB, Divakaran K, Brunengraber LL, Hines RN, McCarver DG (2003). Human hepatic CYP2E1 expression during development. J Pharmacol Exp Ther.

[b13] Koukouritaki SB, Manro JR, Marsh SA, Stevens JC, Rettie AE, McCarver DG (2004). Developmental expression of human hepatic CYP2C9 and CYP2C19. J Pharmacol Exp Ther.

[b14] Hines RN, McCarver DG (2002). The ontogeny of human drug-metabolizing enzymes: phase I oxidative enzymes. J Pharmacol Exp Ther.

[b15] Maruyama M, Matsunaga T, Harada E, Ohmori S (2007). Comparison of basal gene expression and induction of CYP3As in HepG2 and human fetal liver cells. Biol Pharm Bull.

[b16] Yu Y, Zhang C, Zhou G, Wu S, Qu X, Wei H (2001). Gene expression profiling in human fetal liver and identification of tissue- and developmental-stage-specific genes through compiled expression profiles and efficient cloning of full-length cDNAs. Genome Res.

[b17] Ying W, Jiang Y, Guo L, Hao Y, Zhang Y, Wu S (2006). A dataset of human fetal liver proteome identified by subcellular fractionation and multiple protein separation and identification technology. Mol Cell Proteomics.

[b18] Moghe PV, Berthiaume F, Ezzell RM, Toner M, Tompkins RG, Yarmush ML (1996). Culture matrix configuration and composition in the maintenance of hepatocyte polarity and function. Biomaterials.

[b19] Brophy CM, Luebke-Wheeler JL, Amiot BP, Khan H, Remmel RP, Rinaldo P (2009). Rat hepatocyte spheroids formed by rocked technique maintain differentiated hepatocyte gene expression and function. Hepatology.

[b20] Tostoes R, Leite SB, Serra M, Jensen J, Bjorquist P, Carrondo M (2012). Human liver cell spheroids in extended perfusion bioreactor culture for repeated dose drug testing. Hepatology.

[b21] Brandenburger M, Wenzel J, Bogdan R, Richardt D, Nguemo F, Reppel M (2012). Organotypic slice culture from human adult ventricular myocardium. Cardiovasc Res.

[b22] Gauvin R, Larouche D, Marcoux H, Guignard R, Auger FA, Germain L (2012). Minimal contraction for tissue-engineered skin substitutes when matured at the air-liquid interface. J Tissue Eng Regen Med.

[b23] Hanley KP, Oakley F, Sugden S, Wilson DI, Mann DA, Hanley NA (2008). Ectopic SOX9 mediates extracellular matrix deposition characteristic of organ fibrosis. J Biol Chem.

[b24] Carpentier R, Suner RE, Van Hul N, Kopp JL, Beaudry JB, Cordi S (2011). Embryonic ductal plate cells give rise to cholangiocytes, periportal hepatocytes and adult liver progenitor cells. Gastroenterology.

[b25] Furuyama K, Kawaguchi Y, Akiyama H, Horiguchi M, Kodama S, Kuhara T (2011). Continuous cell supply from a Sox9-expressing progenitor zone in adult liver, exocrine pancreas and intestine. Nat Genet.

[b26] Raynaud P, Tate J, Callens C, Cordi S, Vandersmissen P, Carpentier R (2011). A classification of ductal plate malformations based on distinct pathogenic mechanisms of biliary dysmorphogenesis. Hepatology.

[b27] Teh LK, Bertilsson L (2012). Pharmacogenomics of CYP2D6: Molecular genetics, interethnic differences and clinical importance. Drug Metab Pharmacokinet.

[b28] Melton AC, Yee HF (2007). Hepatic stellate cell protrusions couple platelet-derived growth factor-BB to chemotaxis. Hepatology.

[b29] Consortium CHLPP (2010). First insight into the human liver proteome from PROTEOME(SKY)-LIVER(Hu) 1.0, a publicly available database. J Proteome Res.

[b30] Eastman QM (2010). Mammoth data set from human liver reported. J Proteome Res.

[b31] Zhou C, Simpson K, Lancashire LJ, Walker MJ, Dawson MJ, Unwin RD (2012). Statistical considerations of optimal study design for human plasma proteomics and biomarker discovery. J Proteome Res.

[b32] Pan C, Kumar C, Bohl S, Klingmueller U, Mann M (2009). Comparative proteomic phenotyping of cell lines and primary cells to assess preservation of cell type-specific functions. Mol Cell Proteomics.

[b33] Rowe C, Goldring CE, Kitteringham NR, Jenkins RE, Lane BS, Sanderson C (2010). Network analysis of primary hepatocyte dedifferentiation using a shotgun proteomics approach. J Proteome Res.

[b34] Van Summeren A, Renes J, Bouwman FG, Noben JP, van Delft JH, Kleinjans JC (2011). Proteomics investigations of drug-induced hepatotoxicity in HepG2 cells. Toxicol Sci.

[b35] Slany A, Haudek VJ, Zwickl H, Gundacker NC, Grusch M, Weiss TS (2010). Cell characterization by proteome profiling applied to primary hepatocytes and hepatocyte cell lines Hep-G2 and Hep-3B. J Proteome Res.

[b36] Hay DC, Zhao D, Fletcher J, Hewitt ZA, McLean D, Urruticoechea-Uriguen A (2008). Efficient differentiation of hepatocytes from human embryonic stem cells exhibiting markers recapitulating liver development in vivo. Stem Cells.

[b37] Pikkarainen PH, Raiha NC (1967). Development of alcohol dehydrogenase activity in the human liver. Pediatr Res.

[b38] Stewart MJ, Shean ML, Paeper BW, Duester G (1991). The role of CCAAT/enhancer-binding protein in the differential transcriptional regulation of a family of human liver alcohol dehydrogenase genes. J Biol Chem.

[b39] Pitarque M, Rodriguez-Antona C, Oscarson M, Ingelman-Sundberg M (2005). Transcriptional regulation of the human CYP2A6 gene. J Pharmacol Exp Ther.

[b40] Sakai M, Muramatsu M (2007). Regulation of glutathione transferase P: a tumor marker of hepatocarcinogenesis. Biochem Biophys Res Commun.

[b41] Pochareddy S, Edenberg HJ (2010). Identification of a FOXA-dependent enhancer of human alcohol dehydrogenase 4 (ADH4). Gene.

[b42] Masaki T, Tokuda M, Yoshida S, Nakai S, Morishita A, Uchida N (2005). Comparison study of the expressions of myristoylated alanine-rich C kinase substrate in hepatocellular carcinoma, liver cirrhosis, chronic hepatitis, and normal liver. Int J Oncol.

[b43] Garibaldi F, Cicchini C, Conigliaro A, Santangelo L, Cozzolino AM, Grassi G (2012). An epistatic mini-circuitry between the transcription factors Snail and HNF4alpha controls liver stem cell and hepatocyte features exhorting opposite regulation on stemness-inhibiting microRNAs. Cell Death Differ.

[b44] Santangelo L, Marchetti A, Cicchini C, Conigliaro A, Conti B, Mancone C (2011). The stable repression of mesenchymal program is required for hepatocyte identity: a novel role for hepatocyte nuclear factor 4alpha. Hepatology.

