# Supplementary figures and images for: Proteome-wide analyses of human hepatocytes during differentiation and dedifferentiation
Source: Hepatology. 2013 Jul 1;58(2):799–809. doi: 10.1002/hep.26414 (PMC3842115; doi:10.1002/hep.26414)

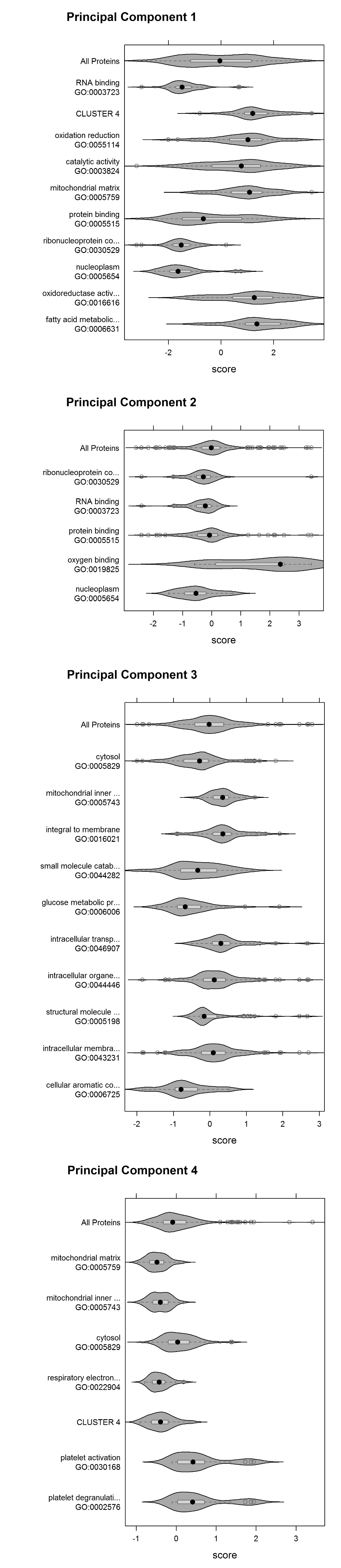

Supplement: Supplementary file 1 [file hep0058-0799-sd1.tif]

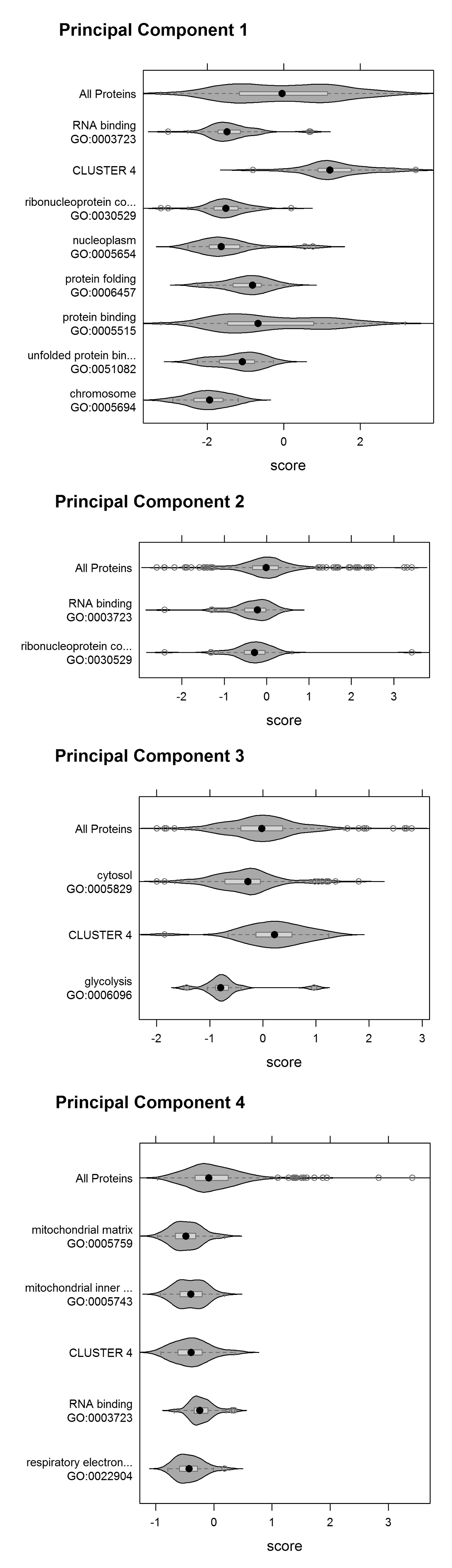

Supplement: Supplementary file 2 [file hep0058-0799-sd2.tif]
