# Supplementary material for: Proteome-wide analyses of human hepatocytes during differentiation and dedifferentiation
Source: Hepatology. 2013 Jul 1;58(2):799–809. doi: 10.1002/hep.26414 (PMC3842115; doi:10.1002/hep.26414)
Supplement: Supplementary file 3 [file hep0058-0799-sd3.doc]

| **Proteins characteristic of fresh human adult hepatocytes: high PC1 score / low PC3 score** | | | | | | |
| --- | --- | --- | --- | --- | --- | --- |
| **I.D.** | **Protein name** | **Abbreviation** | **PC1** | **PC2** | **PC3** | **PC4** |
| P11509 | Cytochrome P450 2A6 | CP2A6_HUMAN | 3.170 | 0.103 | -0.828 | 0.175 |
| Q7Z4W1 | L-xylulose reductase | DCXR_HUMAN | 3.026 | 0.105 | -1.217 | -0.485 |
| P00325 | Alcohol dehydrogenase 1B | ADH1B_HUMAN | 3.003 | 0.021 | -1.368 | -0.009 |
| P00326 | Alcohol dehydrogenase 1C | ADH1G_HUMAN | 2.832 | 0.159 | -1.467 | 0.573 |
| P17516 | Aldo-keto reductase family 1 member C4 | AK1C4_HUMAN | 2.787 | -0.166 | -1.425 | 0.379 |
| O75452 | Retinol dehydrogenase 16 | RDH16_HUMAN | 2.766 | 0.234 | -0.903 | -0.293 |
| P32754 | 4-hydroxyphenylpyruvate dioxygenase | HPPD_HUMAN | 2.712 | -0.081 | -1.851 | -0.196 |
| P08319 | Alcohol dehydrogenase 4 | ADH4_HUMAN | 2.677 | 0.491 | -1.997 | -0.475 |
| O95154 | Aflatoxin B1 aldehyde reductase member 3 | ARK73_HUMAN | 2.311 | -0.238 | -0.770 | 0.112 |
| O95954 | Formimidoyltransferase-cyclodeaminase | FTCD_HUMAN | 2.287 | 0.342 | -0.832 | 0.531 |
| P21695 | Glycerol-3-phosphate dehydrogenase [NAD+], cytoplasmic | GPDA_HUMAN | 2.284 | 0.245 | -1.079 | 0.433 |
| P34896 | Serine hydroxymethyltransferase, cytosolic | GLYC_HUMAN | 2.225 | 0.029 | -0.889 | 0.242 |
| P32929 | Cystathionine gamma-lyase | CGL_HUMAN | 2.189 | 0.072 | -1.818 | -0.199 |
| Q3LXA3 | Bifunctional ATP-dependent dihydroxyacetone kinase/FAD-AMP lyase (cyclizing) | DHAK_HUMAN | 2.091 | -0.177 | -1.453 | 0.014 |
| O75891 | 10-formyltetrahydrofolate dehydrogenase | FTHFD_HUMAN | 1.959 | 0.193 | -0.801 | 0.878 |
| Q16851 | UTP--glucose-1-phosphate uridylyltransferase | UGPA_HUMAN | 1.945 | 0.177 | -0.888 | 0.238 |
| P05181 | Cytochrome P450 2E1 | CP2E1_HUMAN | 1.812 | -0.086 | -0.836 | 0.082 |
| P13929 | Beta-enolase | ENOB_HUMAN | 1.674 | 0.569 | -1.050 | -0.464 |
| P05062 | Fructose-bisphosphate aldolase B | ALDOB_HUMAN | 1.645 | 0.562 | -0.963 | 0.062 |
| Q9UBQ7 | Glyoxylate reductase/hydroxypyruvate reductase | GRHPR_HUMAN | 1.635 | 0.185 | -0.654 | -0.027 |
| P10632 | Cytochrome P450 2C8 | CP2C8_HUMAN | 1.623 | 0.081 | -0.748 | -0.254 |
| P31513 | Dimethylaniline monooxygenase [N-oxide-forming] 3 | FMO3_HUMAN | 1.587 | -0.526 | -0.883 | -0.130 |
| P05089 | Arginase-1 | ARGI1_HUMAN | 1.583 | 0.751 | -0.748 | 0.526 |
| P04424 | Argininosuccinate lyase | ARLY_HUMAN | 1.568 | 0.375 | -0.710 | 0.282 |
| P28332 | Alcohol dehydrogenase 6 | ADH6_HUMAN | 1.518 | 0.779 | -1.437 | -0.169 |
| Q13228 | Selenium-binding protein 1 | SBP1_HUMAN | 1.508 | 0.609 | -0.748 | 0.152 |
| P34913 | Epoxide hydrolase 2 | HYES_HUMAN | 1.492 | 0.612 | -0.675 | -0.211 |
| P11586 | C-1-tetrahydrofolate synthase, cytoplasmic | C1TC_HUMAN | 1.444 | -0.180 | -1.071 | 0.194 |
|  |  |  |  |  |  |  |
| **Proteins characteristic of fresh human fetal hepatocytes: high PC2 score / low PC1 score** | | | | | | |
| **I.D.** | **Protein name** | **Abbreviation** | **PC1** | **PC2** | **PC3** | **PC4** |
| P02792 | Ferritin light chain | FRIL_HUMAN | -1.999 | 2.180 | 0.701 | 0.525 |
| P09211 | Glutathione S-transferase P | GSTP1_HUMAN | -2.007 | 2.142 | 0.120 | 0.563 |
| P16401 | Histone H1.5 | H15_HUMAN | -2.193 | 2.104 | 0.074 | -0.148 |
| P02730 | Band 3 anion transport protein | B3AT_HUMAN | -1.939 | 1.943 | 0.338 | 0.116 |
| P24462 | Cytochrome P450 3A7 | CP3A7_HUMAN | -2.001 | 1.676 | 0.444 | 0.872 |
| P02771 | Alpha-fetoprotein | FETA_HUMAN | -2.723 | 1.672 | -0.061 | 0.306 |
| P08670 | Vimentin | VIME_HUMAN | -1.772 | 1.637 | 1.121 | 1.385 |
| P32119 | Peroxiredoxin-2 | PRDX2_HUMAN | -1.642 | 1.412 | -0.069 | 0.046 |
| Q13907 | Isopentenyl-diphosphate Delta-isomerase 1 | IDI1_HUMAN | -2.168 | 1.326 | 0.110 | 1.104 |
| P16403 | Histone H1.2 | H12_HUMAN | -2.895 | 1.215 | 0.025 | -0.667 |
| P29966 | Myristoylated alanine-rich C-kinase substrate | MARCS_HUMAN | -3.144 | 0.889 | 0.243 | -0.203 |
| Q9Y490 | Talin-1 | TLN1_HUMAN | -1.626 | 0.801 | 0.154 | 0.412 |
| P62805 | Histone H4 | H4_HUMAN | -2.344 | 0.738 | 0.067 | -0.238 |
| P09429 | High mobility group protein B1 | HMGB1_HUMAN | -2.363 | 0.683 | -0.230 | -0.404 |
| P54727 | UV excision repair protein RAD23 homolog B | RD23B_HUMAN | -1.684 | 0.415 | -0.086 | 0.100 |
|  |  |  |  |  |  |  |
| **Proteins characteristic of HepG2 cells: low PC2 score / low PC1 score** | | | | | | |
| **I.D.** | **Protein name** | **Abbreviation** | **PC1** | **PC2** | **PC3** | **PC4** |
| P04075 | Fructose-bisphosphate aldolase A | ALDOA_HUMAN | -2.492 | -2.411 | -0.647 | -0.011 |
| P05455 | Lupus La protein | LA_HUMAN | -3.019 | -2.409 | -0.894 | -0.250 |
| P06733 | Alpha-enolase | ENOA_HUMAN | -1.600 | -1.897 | -0.821 | 0.594 |
| O00299 | Chloride intracellular channel protein 1 | CLIC1_HUMAN | -2.659 | -1.482 | -0.307 | 0.459 |
| P13010 | X-ray repair cross-complementing protein 5 | XRCC5_HUMAN | -1.787 | -1.476 | -0.051 | -0.290 |
| P08238 | Heat shock protein HSP 90-beta | HS90B_HUMAN | -2.257 | -1.447 | -0.683 | -0.109 |
| P06748 | Nucleophosmin | NPM_HUMAN | -2.060 | -1.298 | 0.221 | -0.679 |
| P08727 | Keratin, type I cytoskeletal 19 | K1C19_HUMAN | -1.984 | -1.208 | 0.594 | 0.155 |
| Q07666 | KH domain-containing, RNA-binding, signal transduction-associated protein 1 | KHDR1_HUMAN | -1.697 | -1.188 | 0.076 | -0.186 |
| P19338 | Nucleolin | NUCL_HUMAN | -2.504 | -1.133 | -0.227 | -0.520 |
| P27635 | 60S ribosomal protein L10 | RL10_HUMAN | -1.463 | -1.055 | -0.304 | 0.018 |
| P48643 | T-complex protein 1 subunit epsilon | TCPE_HUMAN | -2.155 | -1.052 | -0.180 | -0.180 |
| Q12905 | Interleukin enhancer-binding factor 2 | ILF2_HUMAN | -1.871 | -1.050 | 0.094 | -0.353 |
| Q00839 | Heterogeneous nuclear ribonucleoprotein U | HNRPU_HUMAN | -1.733 | -0.927 | 0.095 | -0.182 |
| P12956 | X-ray repair cross-complementing protein 6 | XRCC6_HUMAN | -1.961 | -0.908 | -0.086 | -0.101 |
| P50991 | T-complex protein 1 subunit delta | TCPD_HUMAN | -1.581 | -0.851 | -0.197 | 0.039 |
| P62249 | 40S ribosomal protein S16 | RS16_HUMAN | -1.474 | -0.774 | -0.566 | -0.397 |
| P14866 | Heterogeneous nuclear ribonucleoprotein L | HNRPL_HUMAN | -1.843 | -0.753 | 0.084 | -0.190 |
| P07437 | Tubulin beta chain | TBB5_HUMAN | -1.514 | -0.630 | -0.283 | 0.125 |
| P26599 | Polypyrimidine tract-binding protein 1 | PTBP1_HUMAN | -1.931 | -0.608 | -0.141 | -0.081 |
| P40227 | T-complex protein 1 subunit zeta | TCPZ_HUMAN | -1.773 | -0.605 | -0.014 | 0.071 |
| P46783 | 40S ribosomal protein S10 | RS10_HUMAN | -2.228 | -0.589 | -0.408 | -0.317 |
| Q12906 | Interleukin enhancer-binding factor 3 | ILF3_HUMAN | -1.709 | -0.550 | 0.131 | -0.357 |
| P05388 | 60S acidic ribosomal protein P0 | RLA0_HUMAN | -1.451 | -0.533 | -0.246 | -0.355 |
| P52272 | Heterogeneous nuclear ribonucleoprotein M | HNRPM_HUMAN | -1.580 | -0.522 | 0.166 | -0.133 |

*Supporting Table 1. Proteins that discriminated fresh human adult hepatocytes, fetal hepatocytes or HepG2 cells.*

Combinations of scores from the first three principal components (PCs) distinguish all samples of fresh human adult hepatocytes, fetal hepatocytes or HepG2 cells (see Figure 6). For each of the two PCs, the top 75 proteins were identified and proteins common to both lists identified as discriminatory for that particular cell-type. Eight hemoglobins were filtered from the fetal dataset. The first 4 PC scores for the complete dataset are available as Supporting Dataset 3.
