# Supplementary material for: Proteome-wide analyses of human hepatocytes during differentiation and dedifferentiation
Source: Hepatology. 2013 Jul 1;58(2):799–809. doi: 10.1002/hep.26414 (PMC3842115; doi:10.1002/hep.26414)
Supplement: Supplementary file 4 [file hep0058-0799-sd4.doc]

| **Proteins that characterized ALI-3D culture of human adult hepatocytes: high PC4 score / high PC1 score** | | | | | | | |
| --- | --- | --- | --- | --- | --- | --- | --- |
| **I.D.** | **Protein name** | **Abbreviation** | | **PC1** | **PC2** | **PC3** | **PC4** |
| O60218 | Aldo-keto reductase family 1 member B10 | AK1BA_HUMAN | | 1.545 | -1.878 | 1.080 | 3.415 |
| P52895 | Aldo-keto reductase family 1 member C2 | AK1C2_HUMAN | | 1.609 | -0.234 | -0.265 | 2.838 |
| P10909 | Clusterin | CLUS_HUMAN | | 2.126 | -0.569 | 2.705 | 1.874 |
| P04004 | Vitronectin | VTNC_HUMAN | | 1.566 | -0.101 | 2.804 | 1.552 |
| P19971 | Thymidine phosphorylase | TYPH_HUMAN | | 2.291 | -0.077 | -0.118 | 1.396 |
| Q7Z5P4 | 17-beta-hydroxysteroid dehydrogenase 13 | DHB13_HUMAN | | 3.086 | -0.304 | -0.019 | 0.886 |
| O75891 | 10-formyltetrahydrofolate dehydrogenase | FTHFD_HUMAN | | 1.959 | 0.193 | -0.801 | 0.878 |
| P02753 | Retinol-binding protein 4 | RET4_HUMAN | | 2.016 | -0.983 | 1.907 | 0.700 |
| P00326 | Alcohol dehydrogenase 1C | ADH1G_HUMAN | | 2.832 | 0.159 | -1.467 | 0.573 |
| O95954 | Formimidoyltransferase-cyclodeaminase | FTCD_HUMAN | | 2.287 | 0.342 | -0.832 | 0.531 |
| P05089 | Arginase-1 | ARGI1_HUMAN | | 1.583 | 0.751 | -0.748 | 0.526 |
| P22310 | UDP-glucuronosyltransferase 1-4 | UD14_HUMAN | | 3.037 | 0.040 | -0.305 | 0.473 |
| P21695 | Glycerol-3-phosphate dehydrogenase [NAD+], cytoplasmic | GPDA_HUMAN | | 2.284 | 0.245 | -1.079 | 0.433 |
| P07099 | Epoxide hydrolase 1 | HYEP_HUMAN | | 1.948 | 0.212 | 0.099 | 0.398 |
|  |  |  | |  |  |  |  |
| **Proteins that characterized ALI-3D culture of human fetal hepatocytes: high PC4 score / low PC1 score** | | | | | | | |
| **I.D.** | **Protein name** | **Abbreviation** | | **PC1** | **PC2** | **PC3** | **PC4** |
| P08670 | Vimentin | VIME_HUMAN | | -1.772 | 1.637 | 1.121 | 1.385 |
| P08758 | Annexin A5 | ANXA5_HUMAN | | -1.831 | -0.031 | 0.354 | 1.288 |
| Q13907 | Isopentenyl-diphosphate Delta-isomerase 1 | IDI1_HUMAN | | -2.168 | 1.326 | 0.110 | 1.104 |
| P24462 | Cytochrome P450 3A7 | CP3A7_HUMAN | | -2.001 | 1.676 | 0.444 | 0.872 |
| P23528 | Cofilin-1 | COF1_HUMAN | | -1.644 | -0.204 | -0.025 | 0.701 |
| P06733 | Alpha-enolase | ENOA_HUMAN | | -1.600 | -1.897 | -0.821 | 0.594 |
| P09211 | Glutathione S-transferase P | GSTP1_HUMAN | | -2.007 | 2.142 | 0.120 | 0.563 |
| P02792 | Ferritin light chain | FRIL_HUMAN | | -1.999 | 2.180 | 0.701 | 0.525 |
| P29401 | Transketolase | TKT_HUMAN | | -2.009 | -0.246 | -0.258 | 0.496 |
| O00299 | Chloride intracellular channel protein 1 | CLIC1_HUMAN | | -2.659 | -1.482 | -0.307 | 0.459 |
| Q9Y490 | Talin-1 | TLN1_HUMAN | | -1.626 | 0.801 | 0.154 | 0.412 |
|  |  |  | |  |  |  |  |
| **Top 10 proteins that characterized ECM-Sandwich culture of human adult hepatocytes: high PC3 score / high PC1 score** | | | | | | | |
| **I.D.** | **Protein name** | | **Abbreviation** | **PC1** | **PC2** | **PC3** | **PC4** |
| P04004 | Vitronectin | | VTNC_HUMAN | 1.566 | -0.101 | 2.804 | 1.552 |
| P10909 | Clusterin | | CLUS_HUMAN | 2.126 | -0.569 | 2.705 | 1.874 |
| P05783 | Keratin, type I cytoskeletal 18 | | K1C18_HUMAN | 1.058 | -1.107 | 2.674 | -0.183 |
| P05787 | Keratin, type II cytoskeletal 8 | | K2C8_HUMAN | 0.765 | -0.927 | 2.457 | -0.128 |
| P07355 | Annexin A2 | | ANXA2_HUMAN | 0.565 | -0.074 | 1.952 | 1.083 |
| P13473 | Lysosome-associated membrane glycoprotein 2 | | LAMP2_HUMAN | 0.178 | -0.209 | 1.917 | 0.012 |
| P02753 | Retinol-binding protein 4 | | RET4_HUMAN | 2.016 | -0.983 | 1.907 | 0.700 |
| P60660 | Myosin light polypeptide 6 | | MYL6_HUMAN | 0.043 | 0.223 | 1.806 | -0.018 |
| P08684 | Cytochrome P450 3A4 | | CP3A4_HUMAN | 2.608 | -0.585 | 1.529 | 0.361 |
| O00748 | Carboxylesterase 2 | | EST2_HUMAN | 2.331 | -0.635 | 1.484 | -0.041 |

*Supporting Table 2. Proteins that discriminated different culture conditions.*

Combinations of principal component (PC) scores allowed proteins to be identified that were characteristic of ALI-3D and monolayer culture. For ALI-3D culture, for each of the two PCs, the top 75 proteins were identified and proteins common to both lists identified as discriminatory for that particular cell-type. Monolayer culture was distinguished by a high PC3 score and positive PC1 score. The first 4 PC scores for the complete dataset are available as Supporting Dataset 3.
