# Supplementary material for: Proteome-wide analyses of human hepatocytes during differentiation and dedifferentiation
Source: Hepatology. 2013 Jul 1;58(2):799–809. doi: 10.1002/hep.26414 (PMC3842115; doi:10.1002/hep.26414)
Supplement: Supplementary file 9 [file hep0058-0799-sd9.doc]

**Supporting file/figure legends**

**Supporting Dataset 1:** Summary of protein data from four 8-plex iTRAQ experiments quantified relative to a common protein pool. All proteins identified above the 1% global false discovery rate are collated in the 'Protein Summary' sheet and sorted according to the number of samples in which that protein was quantified. The first 432 proteins were detected in each of the 4 iTRAQ experiments and quantified in every sample.

**Supporting Dataset 2:** Enrichment significance of GO terms associated with the 432 proteins expressed and quantified in every sample (commonProteinDetectGO) and in all proteins detected (allProteinDetectGO). Terms were tested using Fisher's exact test, following the elimination algorithm on the GO ontology. Results from Biological Process (BP), Molecular Function (MF) and Cellular Component (CC) ontologies are combined after testing. 'BestCluster' represents the result of functional clustering of annotation terms (see experimental procedures).

**Supporting Dataset 3:** The first 4 principal components scores for each protein identified and quantified in every sample in four iTRAQ experiments.

**Supporting Dataset 4:** Enrichment significance of GO terms (across three ontologies) using the distribution of principal component scores 1-4. Terms were tested using either the Wilcoxon test, the Kolmogorov-Smirnov test or the Wilcoxon test on absolute scores, each following the elimination algorithm on the GO ontology. Results from Biological Process (BP), Molecular Function (MF) and Cellular Component (CC) ontologies are combined after testing. 'BestCluster' represents the result of functional clustering of annotation terms (see Methods). *P* values for all three tests are given.

**Supporting Figure 1** - Violin plots of enriched GO terms for principal components 1-4 (Wilcoxon test). All GO terms with unadjusted *P* value < 1x10^-4^ using the elimination algorithm and the Wilcoxon test on un-transformed PC scores (elimWilcoxon).

**Supporting Figure 2** - Violin plots of enriched GO terms for principal components 1-4 (KS test). All GO terms with unadjusted *P* value < 1x10^-5^ using the elimination algorithm and the Kolmogorov-Smirnov test on PC scores (elimKS).
