# Supplementary material for: Proteome-wide analyses of human hepatocytes during differentiation and dedifferentiation
Source: Hepatology. 2013 Jul 1;58(2):799–809. doi: 10.1002/hep.26414 (PMC3842115; doi:10.1002/hep.26414)
Supplement: Supplementary file 10 [file hep0058-0799-sd10.doc]

### Supporting text

### Human subjects and tissue

##### ***Human adult hepatocytes***

Freshly-isolated cells are considered the highest quality source of human adult hepatocytes , providing a benchmark against which other *in vitro* hepatocyte models can be measured . However, phenotype will inevitably reflect cell handling including factors such as isolation, transport time and conditions. Human adult hepatocytes were sourced commercially from Invitrogen (part of Life Technologies) which obtained surgical resections from living donors as part of a clinical organ donation scheme. As such, the organs had been clinically assessed as free from liver disease and varied in age and BMI from 35-75 years and 17.9-24.1. Three male samples were received and one female sample. Following organ retrieval, cells were prepared overnight in the Invitrogen facility in Warrington, UK, situated 32 kilometres and 28 kilometres from our research facilities in Manchester and Liverpool respectively. As soon as commercial quality assurance measures had been obtained on post-isolation cell viability by trypan blue exclusion, hepatocytes were transported in suspension in proprietary iced transport medium. Transport never lasted more than 1h. On arrival in the laboratory, cells were immediately centrifuged and resuspended twice in cell culture medium (Williams’ E medium supplemented with 2mM L Glutamine, Insulin*-*Transferrin*-*Selenium (ITS), 100nM dexamethasone) and an aliquot taken for cell counting. Viability of an aliquot was determined by trypan blue exclusion and was >90% for each donor. Cells were then processed for protein isolation (for proteomic analysis) or cell culture in Matrigel ‘sandwich’ or ALI-3D format. High attachment efficiency was observed in conventional Matrigel culture with very few cells evident in the media aspirated after 3h when the Matrigel overlay was applied. These cultures were analyzed in duplicate after three days for albumin secretion (mean +/- SEM: 17.95 +/- 6.06 µg/ml) and urea output (211.1 +/- 21.67 µg/ml) to provide further assurance on the reliability of cell quality across samples. The amount of albumin secreted by our cultures compared favourably with other published studies . The cells also readily formed polarized bile canaliculi *in vitro* demonstrated by MRP2 immunoreactivity (Supporting text figure 1).

Taken together, these factors indicate that the fresh human hepatocytes sourced in this study are of high quality.

##### ***Human fetal hepatocytes***

Human fetal samples were collected immediately upon medical or surgical termination of pregnancy by our research nurses and delivered to the research laboratory within 1h. Immediate dissection isolated the liver which was either fixed for immunohistochemistry as previously described or processed for protein isolation or cell culture. The latter was achieved by repeated passage of the tissue gently through the tip of a 1ml pipette until an even cell suspension was acquired. Fig. 3E shows intact fetal liver tissue cultured on ALI-3D membrane for 28 days. The eight samples used for the proteomic and bioinformatic analyses ranged from 8-16 weeks of development.


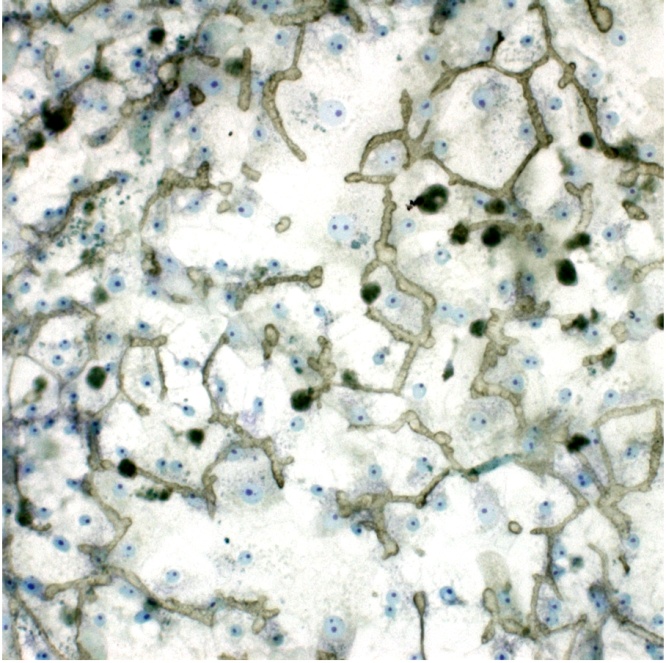


#### Supporting text figure 1. Immunolocalization of MRP2 in Matrigel culture of human adult hepatocytes.

Detection of MRP2 illustrates the formation and association of bile canaliculi as an indicator of hepatocyte phenotype.

#### **Immunohistochemistry and immunoblotting**

Protein lysates were prepared by sonication in triethylammonium bicarbonate buffer (TEAB) /0.1% SDS. Protein concentration was determined by Bradford assay against a BSA standard curve. 10 µg protein lysate was resolved on a 10% polyacrylamide gel and transferred to nitrocellulose membrane for immunoblotting. Membranes were blocked for 1 h in Tris-buffered saline 0.01% Tween 20 (TBS-T) containing 10% milk protein at room temperature. Mouse anti-CYP1A2 (Abcam Ltd), rabbit anti-CYP2D6 (BD Gentest Ltd), rabbit anti-CYP3A4/7 (BD Gentest Ltd), chicken anti-CYP2A6 (gift from Risto Juvonen, University of Eastern Finland), Glutathione S Transferase π (GSTp) (Enzo Life Sciences Ltd) and Rabbit anti HSP47 (Abcam Ltd) were each used at a dilution of 1:5000 in TBS-T with 2% milk protein. Mouse anti-Actin (Abcam Ltd) was used at a dilution of 1:10000. Primary antibodies were incubated overnight at 4°C. Horseradish peroxidise conjugated secondary antibodies (goat anti-rabbit (Sigma Ltd), goat anti-chicken (Sigma Ltd) and rabbit anti-mouse (Sigma Ltd)) were each used at a dilution of 1:5000 in TBS-T with 2% milk protein. Detection was carried out using ECL Plus Western Blotting Detection Reagents and Hyperfilm ECL (Amersham Ltd).

Primary antibodies and dilution used for immunohistochemistry are as follows: rabbit anti-SOX9, Millipore ab5535, at 1:1000; and mouse anti-MRP2, Santa Cruz M2 III-6 sc-59608, at 1:100.

#### **iTRAQ reagent labelling**

Protein isolates were denatured, and cysteine residues were reduced and capped with methylmethanethiosulfate (MMTS) before overnight digestion with trypsin at 37°C and labelling with isobaric tags. A pooled experimental sample was used as a common reference sample to link multiple experiments. iTRAQ-labeled peptides were pooled and diluted to 5 mL with 10 mM potassium dihydrogen phosphate/25% w/v acetonitrile. The pH of the samples was adjusted to < 3 with concentrated phosphoric acid prior to fractionation on a polysulfoethyl A strong cation-exchange column (200 × 4.6 mm, 5 μm, 300 Å; Poly LC, Columbia, MD). A flow rate of 1 mL/min was applied and peptides eluted by increasing the concentration of KCl in the mobile phase to 0.5 M over 60 min. Fractions of 2 mL were collected and were dried by centrifugation under vacuum (SpeedVac, Eppendorf).

LC−MS/MS analysis of iTRAQ-labeled samples was performed on a QSTAR Pulsar I hybrid mass spectrometer (AB Sciex, Warrington, UK). Each cation exchange fraction was resuspended in 120 μL 5% ACN/0.05% trifluoroacetic acid (TFA) and 60 μL loaded on the column and delivered by automated in-line liquid chromatography (integrated LCPackings System, 5 mm C18 nanoprecolumn and 75 μm × 15 cm C18 PepMap column; Dionex, Sunnyvale, CA) via a nanoelectrospray source head and 10 μm inner diameter PicoTip (New Objective, Woburn, MA). The sample was de-salted by washing the precolumn for 30 min at 30 μL/min with 5% ACN/0.05% TFA. A gradient from 5% acetonitrile/0.05% Trifluoroacetic acid (v/v) to 60% acetonitrile /0.05% Trifluoroacetic acid (v/v) was applied at a flow rate of 300 nL/min for 70 min. The MS was operated in positive ion mode with survey scans of 1 s and with an MS/MS accumulation time of 1 s for the three most intense ions. Collision energies were calculated based on the *m*/*z* of the target ion and the formula, collision energy = (slope × *m*/*z*) + the intercept. The intercepts were increased by 3−5 V compared to standard data acquisition to improve the reporter ion intensities/quantitative reproducibility.

Protein identification was performed using ProteinPilot software (Version 3, AB Sciex). The data were analyzed with a fixed modification of MMTS-labeled cysteine, biological modifications allowed, and with the confidence set to 10% to enable the False Discovery Rate to be calculated from screening the reversed Swiss-Prot database. Ratios for each iTRAQ label were obtained relative to the experimental pool which acted as a common reference sample across 4 experimental runs. The detected protein threshold (‘unused protscore (conf)’) in the software was set to 1.3 to achieve 95% confidence.

To avoid inter-experiment bias the adult samples were analyzed across all 4 proteomics experimental runs and the isobaric tag (iTRAQ label) was varied.

#### **Bioinformatics**

##### ***Principal components analysis***

Principal components (PC) analysis (PCA) was performed in R using the set of proteins detected in all samples. Due to the in-experiment normalization of protein intensities, PCA was performed on the covariance matrix without further scaling of protein intensities. The importance of each sample in generating each PC was expressed as a loading. The position of each protein mapped onto each PC was expressed as a score. A small subset of PCs contained a large proportion of the variance in the data. Sample loadings were consistent for samples of the same biological origin.

##### ***GO enrichment - detection test***

The enrichment of gene ontology (GO) terms was tested for using the topGO package in R. The UniProt IDs were mapped to GO terms according to a map hosted by the Gene Ontology project (http://www.geneontology.org/gene-associations/readme/goa.README, created 2011/03/21). Firstly a test for over-representation amongst detected proteins was performed. Both the list of proteins common to all samples and the full list of all detected proteins were used as alternative 'foreground' lists and the full list of Uniprot IDs in the non-redundant GO map were used as the 'background' list. Significant enrichment of individual GO terms was assessed using Fisher's exact test.

##### ***GO enrichment - PC scores test***

Following PCA, GO terms were tested for extreme values of PC scores amongst the group of proteins corresponding to each term. There is no single best method defined for this procedure so three different variations were carried out, each capturing different enrichment signals. In each case the distribution of PC scores was contrasted between a subset of proteins (grouped by a focal GO term) with the background set of other ubiquitous proteins. The Kolmogorov-Smirnov (KS) test, the one-tailed Wilcoxon signed rank test (Wilcoxon) and a Wilcoxon test on absolute PC score values (absWilcoxon) were used. The absWilcoxon test can pick up groups with scores at both extreme ends of the distribution (both very low and very high scores simultaneously) but loses power if the sub-group is truly one-tailed or is distributed asymmetrically about the mean. The test was repeated against each of the PCs 1 to 5.

##### ***GO enrichment - non-independence of terms***

Non-independence of separate GO terms was accounted for in the same way during both the detection and the PC scores tests. As part of the topGO package, the 'elimination' algorithm was used to traverse the GO ontologies (once each for Biological Process (BP), Molecular Function (MF) and Cellular Compartment (CC)), beginning at the lowest level of the ontology (the most specific terms). GO terms assigned to at least n proteins/genes are tested (n=10 was used) and proteins belonging to any significant GO terms are then removed from superior (ancestral) GO terms before those terms are tested. Following the topGO manual, no adjustment was made for multiple testing because there is no null model for the distribution of p-values resulting from the application of the elimination algorithm and the concatenation of results across ontologies. Instead, the p-values were used to rank results and determine candidate proteins and protein groups involved in the processes under study.

##### ***GO enrichment - Functional Clustering***

For presentation, but not testing, we grouped GO terms which, within our dataset, were represented by a very similar set of proteins. Additional overlaps in protein sets occur between distant GO terms both within an ontology category and between GO terms from different ontologies. For example, 90% of the proteins annotated to Biological Process GO:0055114 - 'oxidation reduction' are also annotated to Molecular Function GO:0016491 - 'oxidoreductase activity'. The fuzzy functional clustering of proteins using shared GO annotations was implemented as described in Huang et al. . This algorithm builds functional groups from seed clusters and allows each protein to be a member of any number of functional groups. Seed clusters were created of at least 5 proteins where 50% of members had kappa values of at least 0.65 with each other. Seed clusters were combined where at least 50% of the smaller cluster was contained within a larger cluster. For presentation, where a functional cluster contained 80% of the proteins annotated to a GO term, that GO term was assigned to the functional cluster.

#### **Supporting references**

1. Stephenne X, Najimi M, Sokal EM. Hepatocyte cryopreservation: is it time to change the strategy? World J Gastroenterol;16:1-14.

2. Soars MG, McGinnity DF, Grime K, Riley RJ. The pivotal role of hepatocytes in drug discovery. Chem Biol Interact 2007;168:2-15.

3. Baxter MA, Rowe C, Alder J, Harrison S, Hanley KP, Park BK, Kitteringham NR, et al. Generating hepatic cell lineages from pluripotent stem cells for drug toxicity screening. Stem Cell Res 2010;5:4-22.

4. LeCluyse EL. Human hepatocyte culture systems for the in vitro evaluation of cytochrome P450 expression and regulation. Eur J Pharm Sci 2001;13:343-368.

5. George J, Goodwin B, Liddle C, Tapner M, Farrell GC. Time-dependent expression of cytochrome P450 genes in primary cultures of well-differentiated human hepatocytes. J Lab Clin Med 1997;129:638-648.

6. Kono Y, Yang S, Roberts EA. Extended primary culture of human hepatocytes in a collagen gel sandwich system. In Vitro Cell Dev Biol Anim 1997;33:467-472.

7. Dvorak Z, Ulrichova J, Pichard-Garcia L, Modriansky M, Maurel P. Comparative effect of colchicine and colchiceine on cytotoxicity and CYP gene expression in primary human hepatocytes. Toxicol In Vitro 2002;16:219-227.

8. Piper K, Brickwood S, Turnpenny LW, Cameron IT, Ball SG, Wilson DI, Hanley NA. Beta cell differentiation during early human pancreas development. J Endocrinol 2004;181:11-23.

9. Huang da W, Sherman BT, Tan Q, Collins JR, Alvord WG, Roayaei J, Stephens R, et al. The DAVID Gene Functional Classification Tool: a novel biological module-centric algorithm to functionally analyze large gene lists. Genome Biol 2007;8:R183.
